# Aspirin, clopidogrel and prasugrel monotherapy in patients with type 2 diabetes mellitus: a double-blind randomised controlled trial of the effects on thrombotic markers and microRNA levels

**DOI:** 10.1186/s12933-019-0981-3

**Published:** 2020-01-07

**Authors:** William A. E. Parker, Christian Schulte, Temo Barwari, Fladia Phoenix, Sam M. Pearson, Manuel Mayr, Peter J. Grant, Robert F. Storey, Ramzi A. Ajjan

**Affiliations:** 1 Department of Infection, Immunity & Cardiovascular Disease, University of Sheffield, Sheffield, UK; 2 King’s British Heart Foundation Centre, King’s College London, London, UK; 3 Department of General and Interventional Cardiology, University Heart Centre Hamburg Eppendorf, Hamburg, Germany; 4 Leeds Institute of Cardiovascular and Metabolic Medicine, University of Leeds, Leeds, UK

**Keywords:** Aspirin, Diabetes mellitus, Micro RNA, P2Y_12_ inhibitors, Platelet inhibition

## Abstract

**Background:**

Despite increased atherothrombotic risk in type 2 diabetes mellitus, (T2DM) the best preventative antithrombotic strategy remains undetermined. We defined the effects of three antiplatelet agents on functional readout and biomarker kinetics in platelet activation and coagulation in patients with T2DM.

**Materials and methods:**

56 patients with T2DM were randomised to antiplatelet monotherapy with aspirin 75 mg once daily (OD), clopidogrel 75 mg OD or prasugrel 10 mg OD during three periods of a crossover study. Platelet aggregation (PA) was determined by light-transmittance aggregometry and P-selectin expression by flow cytometry. Markers of fibrin clot dynamics, inflammation and coagulation were measured. Plasma levels of 14 miRNA were assessed by quantitative polymerase chain reactions.

**Results:**

Of the 56 patients, 24 (43%) were receiving aspirin for primary prevention of ischaemic events and 32 (57%) for secondary prevention. Prasugrel was the strongest inhibitor of ADP-induced PA (mean ± SD maximum response to 20μmol/L ADP 77.6 ± 8.4% [aspirin] vs. 57.7 ± 17.6% [clopidogrel] vs. 34.1 ± 14.1% [prasugrel], p < 0.001), P-selectin expression (30 μmol/L ADP; 45.1 ± 21.4% vs. 27.1 ± 19.0% vs. 14.1 ± 14.9%, p < 0.001) and collagen-induced PA (2 μg/mL; 62.1 ± 19.4% vs. 72.3 ± 18.2% vs. 60.2 ± 18.5%, p < 0.001). Fibrin clot dynamics and levels of coagulation and inflammatory proteins were similar. Lower levels of miR-24 (p = 0.004), miR-191 (p = 0.019), miR-197 (p = 0.009) and miR-223 (p = 0.014) were demonstrated during prasugrel-therapy vs. aspirin. Circulating miR-197 was lower in those cardiovascular disease during therapy with aspirin (p = 0.039) or prasugrel (p = 0.0083).

**Conclusions:**

Prasugrel monotherapy in T2DM provided potent platelet inhibition and reduced levels of a number of platelet-associated miRNAs. miR-197 is a potential marker of cardiovascular disease in this population. Clinical outcome studies investigating prasugrel monotherapy are warranted in individuals with T2DM.

*Trial registration* EudraCT, 2009-011907-22. Registered 15 March 2010, https://www.clinicaltrialsregister.eu/ctr-search/trial/2009-011907-22/GB.

## Background

Thrombotic events are associated with a large burden of morbidity and mortality in the general population [1], with an even higher risk in patients with type 2 diabetes mellitus (T2DM) [2]. Enhanced platelet activation and altered fibrin clot properties are central pathological processes in the development of thrombosis in T2DM, thus increasing the risk of cardiovascular events and contributing to adverse clinical outcome following vascular ischaemia [3–5].

Antiplatelet drugs for the treatment and prevention of atherothrombosis have largely focussed on two pathways of platelet activation: thromboxane A_2_ generation, which is blocked by the irreversible cyclo-oxygenase inhibitor aspirin, and adenosine diphosphate- (ADP-)induced amplification of platelet activation via the P2Y_12_ receptor, which is irreversibly inhibited by thienopyridines such as clopidogrel and prasugrel [6]. In combination with aspirin, thienopyridines reduce the risk of thrombotic events after acute coronary syndrome (ACS) [7], but the protective effects can vary according to the agent used and the population studied. Following coronary ischaemia requiring percutaneous intervention, prasugrel has shown enhanced vascular protective properties compared with clopidogrel in T2DM patients without an increase in bleeding risk, in contrast to individuals without T2DM [8, 9].

In the clopidogrel vs aspirin in patients at risk of ischemic events (CAPRIE) study, clopidogrel, used as single antiplatelet therapy (SAPT) showed better protection against vascular ischaemia compared with aspirin monotherapy in patients with T2DM [10], an effect that was even more pronounced in insulin-treated subjects [11]. In contrast to clopidogrel, prasugrel as SAPT has not been well studied and data on patients with T2DM are scarce.

Studies of aspirin for the primary prevention of cardiovascular disease in patients T2DM have been disappointing, with little or no reduction in vascular ischaemic events at the expense of a significant increase in bleeding risk [12–16]. Similarly, the pharmacokinetics and clinical efficacy of clopidogrel, a pro-drug, display significant variability between individuals due to differences in activity of cytochrome P450 2C19 [17]. Prasugrel, whilst also a pro-drug, is activated by a different, more predictable metabolic pathway and therefore offers better inter-individual consistency of effect [18].

In addition to the effects on platelets, aspirin and P2Y_12_ inhibitors may modulate the fibrin network [19, 20] and affect vascular inflammatory pathways [21, 22]. In order to assess a functional readout and to gain further mechanistic insight, platelet function tests have been used to assess the response to antiplatelet agents. Micro-ribonucleic acids (miRNAs) are emerging as an adjunct to our understanding and assessment of platelet function [23–25]. From a translational point of view, detection of a variety of miRNAs has been linked with clinical outcomes including ischaemic heart disease [26].

Prasugrel monotherapy in individuals with T2DM may offer superior anti-thrombotic properties compared with aspirin or clopidogrel. The aim of this study was to comprehensively characterise and compare the effects of the three drugs on platelet function, fibrin network characteristics, inflammation and expression of miRNAs in a cohort of T2DM patients.

## Methods

### Study design

**Fig. 1:**
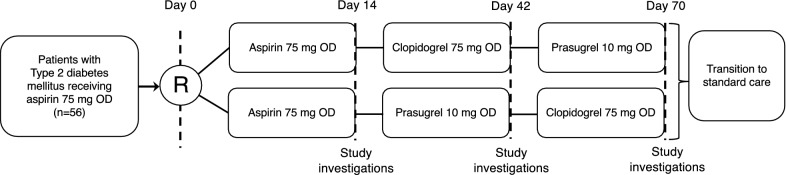
Design of the study. *mg* milligrams, *OD* once daily, *R* point of randomisation

We performed a single-centre, double-blind, crossover, randomised controlled trial of patients with a confirmed diagnosis of T2DM. Patients were eligible to participate if they were aged 18–75 years, already on treatment with aspirin 75 mg once-daily (OD) and able to give informed consent. Eligible participants receiving aspirin 75 mg OD were randomised 1:1 to one of two medication sequences (Fig. 1). All patients continued aspirin 75 mg OD for an initial lead-in period of 14 days. One half then received clopidogrel 75 mg OD for 28 days then prasugrel 10 mg OD for 28 days, whilst the other half received prasugrel 10 mg OD for 28 days followed by clopidogrel 75 mg OD for 28 days. Aspirin was discontinued after study completion if it was deemed clinically unnecessary. Randomisation was performed by shuffled sealed opaque envelopes prepared by a member of pharmacy staff. Both patients and investigators were blind to treatment allocation until all study data was collected. A range of pharmacodynamic measurements were made at the end of each treatment period. The study was approved by the United Kingdom National Health Service Research Ethics Service (reference 09/H1307/110). Written consent was obtained from participants before any study activities took place.

Exclusion criteria included: any type of diabetes other than T2DM; any coagulation disorder; neoplastic disease; history of ACS within 3 months of enrolment; history of stroke or transient ischaemic attack; history of deep venous thrombosis or pulmonary embolism; treatment with oral anticoagulant or non-steroidal anti-inflammatory drugs; abnormal liver enzyme tests defined as alanine transferase > threefold upper limit of normal; any previous or current upper gastrointestinal pathology; weight < 60 kg; and women of child-bearing age and refusing to use contraception.

### Blood samples

Venous blood samples were collected by venepuncture, using syringe and 18G needle, and anticoagulated with trisodium citrate dihydrate 3.13%. Platelet-rich plasma was prepared by centrifugation for 10 min at 200×*g* and platelet-poor plasma was prepared by further centrifugation for 10 min at 1500×*g*. All analyses described below were undertaken by individuals blinded to the sample details and type of antiplatelet treatment.

### Platelet aggregation

Light transmittance aggregometry (LTA) was performed using ADP (1, 2, 5, 10 and 20 µmol/L), arachidonic acid (AA, 1.0 mmol/L) and collagen (2 and 16 µg/mL) as agonists and a PAP-8 aggregometer (v2.0, Bio/Data Corporation, Horsham, PA, USA), as previously described [27]. Maximum (MA) and final (FA) aggregation responses at 6 min after agonist injection, adjusted for baseline, were recorded. Samples were assessed in duplicate, taking the mean value for analysis, and repeated if a discrepancy of > 10% was observed between the readings.

### Platelet P-selectin expression

Surface expression of platelet P-selectin after stimulation with 0.3, 1, 3, 10 & 30 μmol/L ADP was quantified using flow cytometry and expressed as percentage positive events, as previously described [28].

### Fibrin clot turbidimetric analysis

High-throughput turbidimetric analysis was performed as previously described and validated [29–32]. Briefly, citrated platelet-poor plasma samples were mixed with standard lysis and activation mixes to form acellular clots. Serial absorbance was measured using an automated plate reader during clot formation until lysis was achieved. Variables recorded were lag time, representing the period from the addition of clot activation mix to the start of clot formation (a measure of clotting tendency), final clot turbidity (maximum absorbance, a representation of fibre thickness and clot density), and lysis time (time from full clot formation to 50% lysis, a measure of fibrinolysis potential).

### Coagulation-associated proteins and inflammatory markers

Levels of plasminogen activator inhibitor 1 (PAI1) were determined in citrated plasma using enzyme-linked immunosorbent assay, as previously described [32]. Fibrinogen levels were determined by the clotting method of Clauss using a KC 10TM coagulometer (Henrich Amelung GmbH, Lemgo, Germany), as described elsewhere [33, 34]. Levels of C-reactive protein (CRP) and complement C3 were determined, as previously described [31].

### RNA isolation and miRNA quantification

Quantification of miRNAs with known relevance to platelet function and cardiovascular disease (miRs 21, 24, 27b, 28, 93, 122, 126, 150, 191, 197, 223, 320, 451a and 486) was performed in platelet-poor plasma samples using reverse transcription quantitative polymerase chain reaction (RT-qPCR), as previously described [24, 26]. Total RNA was isolated using the miRNeasy Mini kit (Qiagen, Hilden, Germany). In brief, 100 µL of plasma were combined with 694.75 µL of QIAzol, 4 µL of diluted *Caenorhabditis elegans* miR-39-3p (cel-miR-39) spike-in and 1.25 µL carrier MS2. Following a brief incubation at ambient temperature, 140 µL of chloroform were added and the solution was mixed vigorously. Samples were then centrifuged at 13,500 relative centrifugal force (rcf) for 15 min at 4 °C. The upper aqueous phase was carefully transferred to a new tube and 1.5 volumes of ethanol were added. The samples were then applied directly to columns and washed according to the company’s protocol. Total RNA was eluted with 35 µL of nuclease-free water. A fixed volume of 3 μL of the 35μL RNA eluate was used as input for reverse transcription (RT) reactions. MiRNAs were reverse-transcribed using Megaplex RT Primer Pools (Human Pool A version 2.1; Life Technologies, Darmstadt, Germany) and the TaqMan MicroRNA RT kit (Life Technologies, Darmstadt, Germany) according to the manufacturer’s instructions. Templates were pre-amplified using Megaplex PreAmp Primers (Primers A version 2.1) and PreAmp Mastermix (Life Technologies) with 12 cycles of 95 °C for 15 s and 60 °C for 4 min. Pre-amplification product was 72 times diluted and 2.25 μL were combined with 0.25 μL TaqMan microRNA assay (20×) (Life Technologies) and 2.5 μL TaqMan Universal PCR Master Mix No AmpErase UNG (2×) to a final volume of 5 μL. RT-qPCR was performed on an Applied Biosystems Viia 7 thermocycler at 95 °C for 10 min, followed by 40 cycles of 95 °C for 15 s and 60 °C for 1 min. Clinical data were blinded to laboratory personnel.

Cel-miR-39 was used for normalisation and as a quality control. Quantification results were calibrated with pooled RNA from 50 samples. Quantification cycle (Cq) values > 32 were considered to fall below the limit of detection. Relative quantification was performed with Microsoft Excel, version 15.32 for Mac using the 2^(−∆∆Cq)^ method [35].

### Statistical analysis

The three treatments were compared by repeated measures ANOVA with Greenhouse–Geisser correction. The primary endpoints of the study were platelet aggregation responses to ADP, collagen and AA; platelet P-selectin expression and fibrin clot dynamics. Other analyses were exploratory. For variables with a significant difference (p < 0.05) between the treatments, pairwise comparisons were performed using Bonferroni correction. SPSS statistics v25 (IBM software) was used for these analyses and graphical representations generated using GraphPad PRISM v7. Correlation and subgroup analyses were performed using RStudio v1.1.456: adjustment for multiple comparisons was not made for these as they were exploratory and intended for hypothesis generation.

A total of 56 patients were needed to detect a 10% difference in platelet aggregation response to various agonists comparing the different therapies at p < 0.05 and 90% power, based on the assumption that the common standard deviation of the response variable is 16%. The study also had the power to detect a 7% difference in clot final turbidity based on the assumption that standard deviation for the response variable is 11% (p < 0.05, 90% power).

## Results

### Recruitment and participant characteristics

Of 310 patients who were approached, 64 were enrolled and 56 completed the study (Additional file 1: Figure S1). Baseline characteristics are shown in Table 1.

**Table 1:** Baseline characteristics of participants completing the study

	Participants (n = 56)
Sex	
Female/male	9/47
Age (years)	
Mean (range)	60.7 (46–73)
Smoking (Y/N)	11/45
Blood pressure [mean ± SD]	
Systolic (mmHg)	132.0 ± 16.32
Diastolic (mmHg)	80.0 ± 9.51
Physical examination [mean ± SD]	
Height (m)	1.72 ± 0.07
Weight (kg)	96.18 ± 17.60
BMI	32.70 ± 5.23
Baseline blood tests [mean ± SD]	
HbA1c (mmol/mol)	71.60 ± 22.99
Sodium (mmol/L)	139.28 ± 2.39
Potassium (mmol/L)	4.48 ± 0.39
Creatinine (μmol/L)	83.13 ± 20.66
Urea (mmol/L)	6.20 ± 2.41
eGFR (mL/min/1.73 m^2^)	80.70 ± 13.24
Total cholesterol (mmol/L)	3.79 ± 0.77
LDL (mmol/L)	1.89 ± 0.49
HDL (mmol/L)	1.08 ± 0.26
Triglycerides (mmol/L)	2.06 ± 2.01
FT_4_ (pmol/L)	15.10 ± 2.12
TSH (mIU/L)	2.00 ± 1.12
History of macrovascular disease	32 (57%)
Microvascular complications	
Retinopathy	23 (41%)
Nephropathy	9 (16%)
Neuropathy	15 (27%)
Concomitant medication and therapies	
Diabetes related	
Metformin	48 (86%)
Sulphonylurea	15 (27%)
Gliptin	6 (11%)
Glitazone	5 (9%)
GLP-1 analogues	5 (9%)
SGLT2-inhibitors	0 (0%)
Insulin	27 (48%)
Antihypertensives	
ACE inhibitor/ARB	48 (86%)
Calcium channel blocker	13 (23%)
Diuretic	16 (29%)
Beta-blockers	26 (47%)
Αlpha-blockers	5 (9%)
Lipid-lowering	
Statin	52 (93%)
Fibrate	1 (2%)
Ezetimibe	7 (13%)

*ARB* angiotensin receptor blocker, *FT*_*4*_ free thyroxine, *GLP-1* glucagon like peptide 1, *SGLT2* sodium-glucose transporter 2, *TSH* thyroid stimulating hormone

### Platelet aggregation responses

**Fig. 2:**
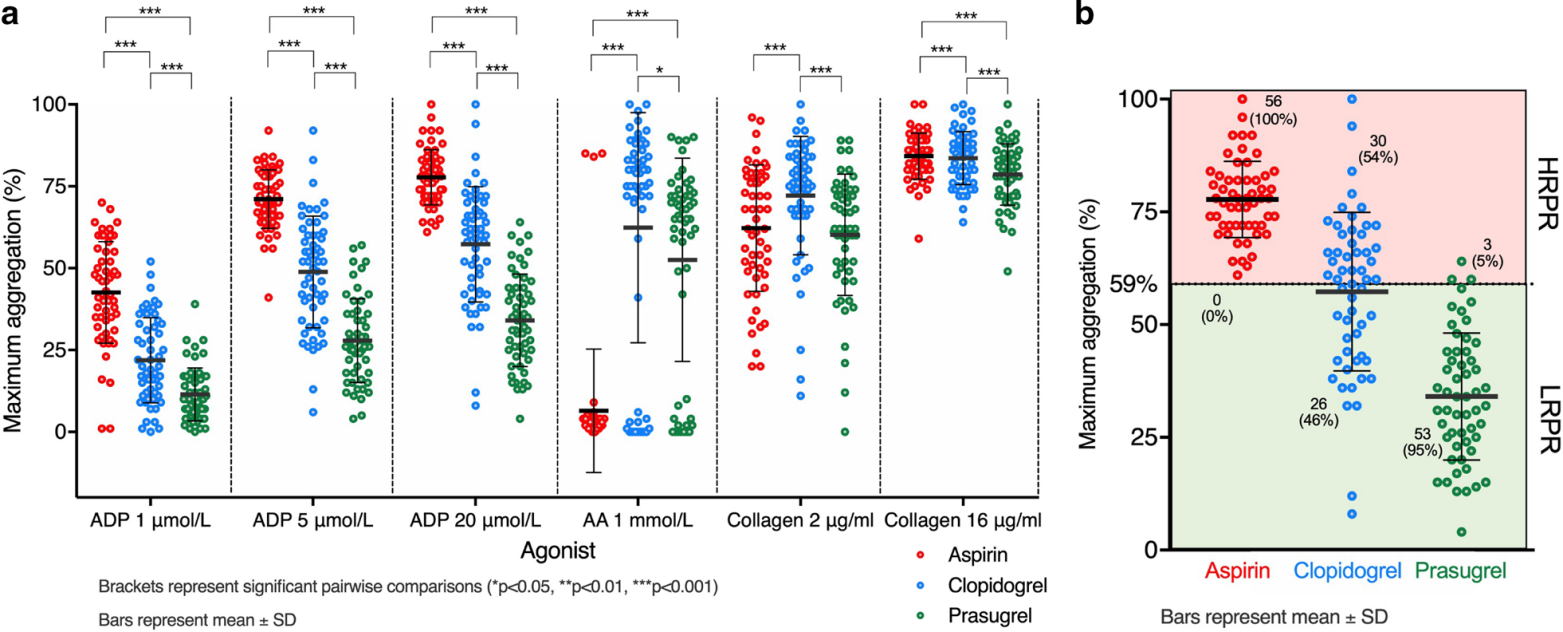
**a** Maximum platelet aggregation responses assessed by light transmittance aggregometry. *AA* arachidonic acid; **b** proportions of participants during each treatment period with high (HRPR) and low residual platelet reactivity (LRPR), defined as a maximum aggregation response to 20 μmol/L ADP of > 59% and ≤ 59% respectively

Maximum aggregation (MA) responses are summarised in Tables 2, 3 and Fig. 2a. Between the 3 treatments, there were significant differences in MA responses to all the agonists and concentrations tested (all p < 0.001). ADP-induced platelet aggregation, at all 5 concentrations tested, was significantly greater when receiving aspirin compared to clopidogrel (all p < 0.001) and clopidogrel compared to prasugrel (all p < 0.001). In contrast, platelet aggregation responses to 1 mmol/L AA were significantly lower when receiving aspirin (6.6 ± 19.0%) compared to clopidogrel (63.4 ± 34.6%, p < 0.001) and prasugrel (52.6% ± 31.1%, p < 0.001). The difference between clopidogrel and prasugrel was also significant (p = 0.027). The response to collagen 2 μg/mL was significantly reduced when receiving aspirin (62.1 ± 19.4%) compared to clopidogrel (72.3 ± 18.2%, p = 0.001), while prasugrel-treated individuals had a similar response to those on aspirin (60.2 ± 18.5%, p > 0.99). The response to collagen 16 μg/mL was similar when receiving aspirin (84.4 ± 7.0%) compared to clopidogrel (83.8 ± 8.1%, p > 0.99) but was lower when receiving prasugrel (78.6 ± 9.4%, p < 0.001). Compared to prasugrel, responses to both 2 and 16 μg/mL collagen were more pronounced when receiving clopidogrel (p < 0.001 and 0.003 respectively). FA responses followed a similar pattern (data not shown).

**Table 2:** Results of study endpoints relating to platelet function, P-selectin expression, fibrin clot dynamics, coagulation-associated proteins and inflammatory markers

		Aspirin (n = 56)	Clopidogrel (n = 56)	Prasugrel (n = 56)	p
		Mean ± SD	Mean ± SD	Mean ± SD	
Maximum platelet aggregation response (%)					
*Agonist*	*[Agonist]*				
ADP	1 μmol/L	42.7 ± 15.6	22.3 ± 12.9	11.5 ± 8.1	< 0.001
	2 μmol/L	61.0 ± 12.4	37.9 ± 17.0	21.0 ± 11.4	< 0.001
	5 μmol/L	71.0 ± 8.9	49.2 ± 17.0	27.9 ± 12.8	< 0.001
	10 μmol/L	80.5 ± 9.7	58.2 ± 18.1	33.6 ± 14.3	< 0.001
	20 μmol/L	77.6 ± 8.4	57.7 ± 17.6	34.1 ± 14.1	< 0.001
AA	1 mmol/L	6.6 ± 19.0	63.4 ± 34.6	52.6 ± 31.1	< 0.001
Collagen	2 μg/mL	62.1 ± 19.4	72.3 ± 18.2	60.2 ± 18.5	< 0.001
	16 μg/mL	84.4 ± 7.0	83.8 ± 8.1	78.6 ± 9.4	< 0.001
Platelet P-selectin expression (%)					
*[ADP]* (μmol/L)					
0.3		18.4 ± 13.0	12.5 ± 10.4	5.9 ± 5.0	< 0.001
1		31.2 ± 19.0	18.2 ± 14.5	9.0 ± 6.8	< 0.001
3		37.8 ± 23.1	23.5 ± 17.3	10.4 ± 7.7	< 0.001
10		41.5 ± 23.7	24.2 ± 17.6	11.5 ± 8.1	< 0.001
30		45.1 ± 21.4	27.1 ± 19.0	14.1 ± 14.9	< 0.001
Fibrin clot dynamics					
Lag time	(s)	683.3 ± 170.4	706.9 ± 196.2	665.0 ± 151.8	0.007
Maximum absorbance	(AU)	0.2 ± 0.08	0.2 ± 0.09	0.2 ± 0.08	0.65
Lysis time	(s)	519.6 ± 112.3	522.3 ± 132.8	522.4 ± 101.2	0.95
Coagulation-associated proteins					
Fibrinogen	(g/L)	2.8 ± 0.5	2.7 ± 0.5	2.7 ± 0.5	0.69
PAI-1	(pg/mL)	2334.6 ± 1675.3	2132.5 ± 1626.4	2089.3 ± 1667.1	0.27
Inflammatory markers					
WCC	(×10^6^/L)	7.2 ± 1.8	6.7 ± 1.9	7.0 ± 2.0	0.067
CRP	(mg/L)	2.3 ± 3.4	2.4 ± 4.6	1.8 ± 2.2	0.5
C3	(g/L)	0.7 ± 0.1	0.7 ± 0.1	0.7 ± 0.1	0.27

p values represent a repeated-measures ANOVA conducted between the three treatments

*AA* arachidonic acid, *C3* complement fragment 3, *PAI-1* plasminogen activator inhibitor 1, *WCC* white cell count

**Table 3:** Pairwise comparisons (with Bonferroni correction) for those endpoints with significant differences between the three treatments on ANOVA

		Aspirin vs. clopidogrel		Aspirin vs. prasugrel		Clopidogrel vs. prasugrel	
		Mean difference (95% CI)	p	Mean difference (95% CI)	p	Mean difference (95% CI)	p
Maximum platelet aggregation response (%)							
*Agonist*	*Concentration*						
ADP	1 μmol/L	20.46 (16.33 to 24.58)	< 0.001	31.24 (26.79 to 35.68)	< 0.001	10.78 (7.34 to 14.22)	< 0.001
	2 μmol/L	23.04 (17.17 to 29.91)	< 0.001	39.96 (35.75 to 44.18)	< 0.001	16.93 (12.48 to 21.00)	< 0.001
	5 μmol/L	21.80 (16.01 to 27.59)	< 0.001	43.07 (38.54 to 47.60)	< 0.001	21.27 (16.75 to 25.80)	< 0.001
	10 μmol/L	22.29 (15.97 to 28.61)	< 0.001	46.86 (41.39 to 52.32)	< 0.001	24.56 (28.98 to 20.14)	< 0.001
	20 μmol/L	19.94 (13.71 to 26.14)	< 0.001	43.53 (38.57 to 48.49)	< 0.001	23.60 (19.34 to 27.83)	< 0.001
AA	1 mmol/L	− 56.86 (− 68.89 to − 44.82)	< 0.001	− 45.98 (− 56.77 to − 35.20)	< 0.001	10.87 (0.95 to 20.79)	0.027
Collagen	2 μg/mL	− 10.24 (− 16.52 to − 3.95)	0.001	1.86 (− 3.22 to 6.92)	> 0.99	12.09 (7.52 to 16.66)	< 0.001
	16 μg/mL	0.62 (− 2.28 to 3.52)	> 0.99	5.73 (2.71 to 8.74)	< 0.001	5.11 (1.48 to 8.74)	0.003
Platelet P-selectin expression (%)							
*[ADP] μmol/L*							
0.3		5.87 (2.05 to 9.69)	0.001	12.52 (8.32 to 16.72)	< 0.001	6.65 (3.65 to 9.65)	< 0.001
1		13.00 (6.56 to 19.43)	< 0.001	22.23 (16.00 to 28.45)	< 0.001	9.23 (5.30 to 13.16)	< 0.001
3		14.27 (6.95 to 21.58)	< 0.001	27.37 (19.63 to 35.11)	< 0.001	13.10 (8.19 to 18.01)	< 0.001
10		17.28 (9.90 to 24.66)	< 0.001	29.95 (22.17 to 37.74)	< 0.001	12.67 (7.55 to 17.80)	< 0.001
30		18.02 (9.82 to 26.22)	< 0.001	30.97 (23.13 to 38.81)	< 0.001	12.95 (7.22 to 18.68)	< 0.001
Fibrin clot dynamics							
Lag time (s)		− 23.61 (− 56.2 to 8.98)	0.237	18.28 (− 8.57 to 45.12)	0.295	41.8 (7.41 to 76.37)	0.012

*AA* arachidonic acid

Comparing platelet aggregation responses when receiving standard-of-care aspirin 75 mg OD to those at the end of the study aspirin period, there were no differences (Additional file 1: Table S1).

### High residual platelet reactivity

High on-treatment residual platelet reactivity (HRPR), which has been associated with increased ischaemic risk, can be defined based on a maximum aggregatory response to 20 μmol/L ADP of > 59%, when assessed by LTA [36–38]. In this study, whilst receiving aspirin, all participants had HRPR. The proportion was reduced compared to aspirin when receiving either clopidogrel (relative risk [RR] 0.54, 95% CI [0.41–0.66], p < 0.0001) or prasugrel (RR 0.05 [0.02–0.15], p < 0.0001), and when receiving prasugrel compared to clopidogrel (RR 0.1 [0.03–0.28], p < 0.0001) (Additional file 1: Table S2, Fig. 2b).

### Platelet P-selectin expression

**Fig. 3:**
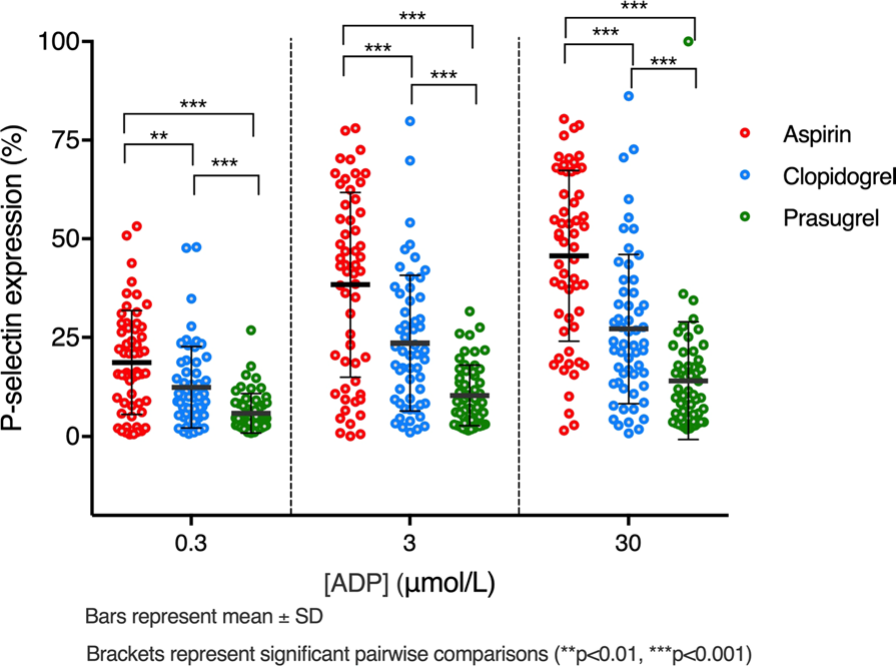
Platelet P-selectin expression in response to stimulation with ADP at concentrations of 0.3, 3 and 30 μmol/L

Measurement of ADP-stimulated platelet P-selectin expression revealed significant differences between the 3 treatments at all concentrations of ADP used (e.g. 30 μmol/L: aspirin 45.1 ± 21.4% vs. clopidogrel 27.1 ± 19.0% vs. prasugrel 14.1 ± 14.9%, p < 0.001) (Table 2, Fig. 3). On pairwise comparison, expression was significantly greater when treated with clopidogrel vs. aspirin, prasugrel vs. aspirin and prasugrel vs. clopidogrel (all p ≤ 0.001) (Table 3, Fig. 3).

### Fibrin clot properties

Lag time was significantly different when comparing aspirin, clopidogrel and prasugrel treatment (683.3 ± 170.4 s vs. 706.9 ± 196.2 s vs. 665.0 ± 151.8 s, respectively, p = 0.007 [ANOVA]) (Table 2, Additional file 1: Figure S1). There was no difference between aspirin and clopidogrel (p = 0.24), nor aspirin and prasugrel (p = 0.30), but lag time was significantly longer when receiving clopidogrel vs. prasugrel (p = 0.012) (Table 3, Additional file 1: Figure S2). There were no significant differences between the treatments in final clot turbidity (0.2 ± 0.08 (arbitrary units) vs. 0.2 ± 0.09 vs. 0.2 ± 0.08, p = 0.65) or lysis time (519.6 ± 112.3 s vs. 522.3 ± 132.8 s vs. 522.4 ± 101.2, p = 0.95) (Table 2, Additional file 1: Figure S3). Lysis time, but not other parameters, significantly correlated with HbA1_c_ (R = 0.18, p = 0.027) (Additional file 1: Table S3).

### Coagulation factors and inflammatory markers

No significant differences in fibrinogen, circulating leukocyte count, CRP or complement C3 were observed between the treatments (all p > 0.05, Table 2).

### MiRNA quantification

**Fig. 4:**
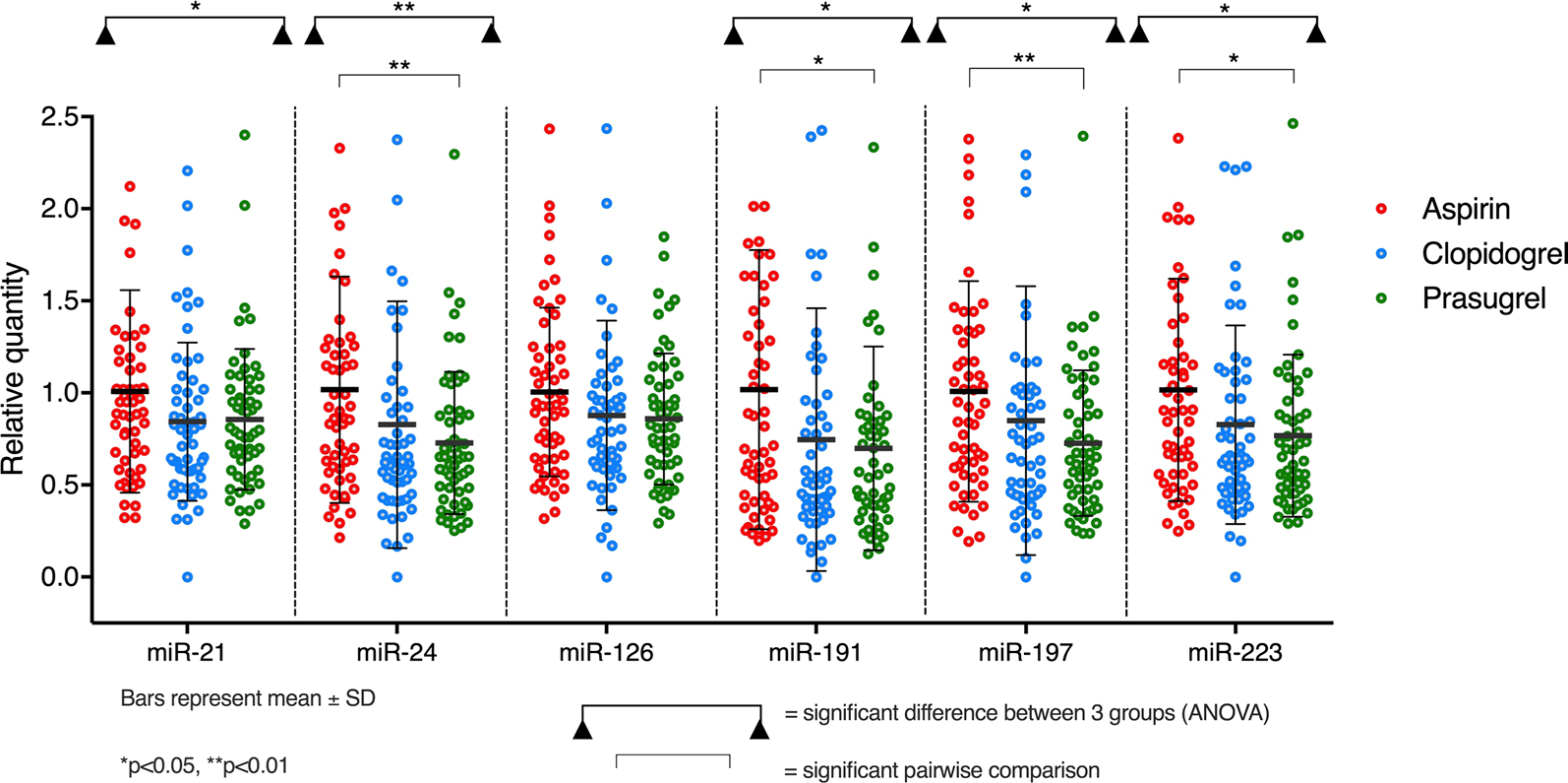
Quantification of plasma levels of miR-21, miR-24, miR-126, miR-191, miR-197 and miR-223 using the 2^−ΔΔcq^ method, expressed relative to the mean value when receiving aspirin

**Fig. 5:**
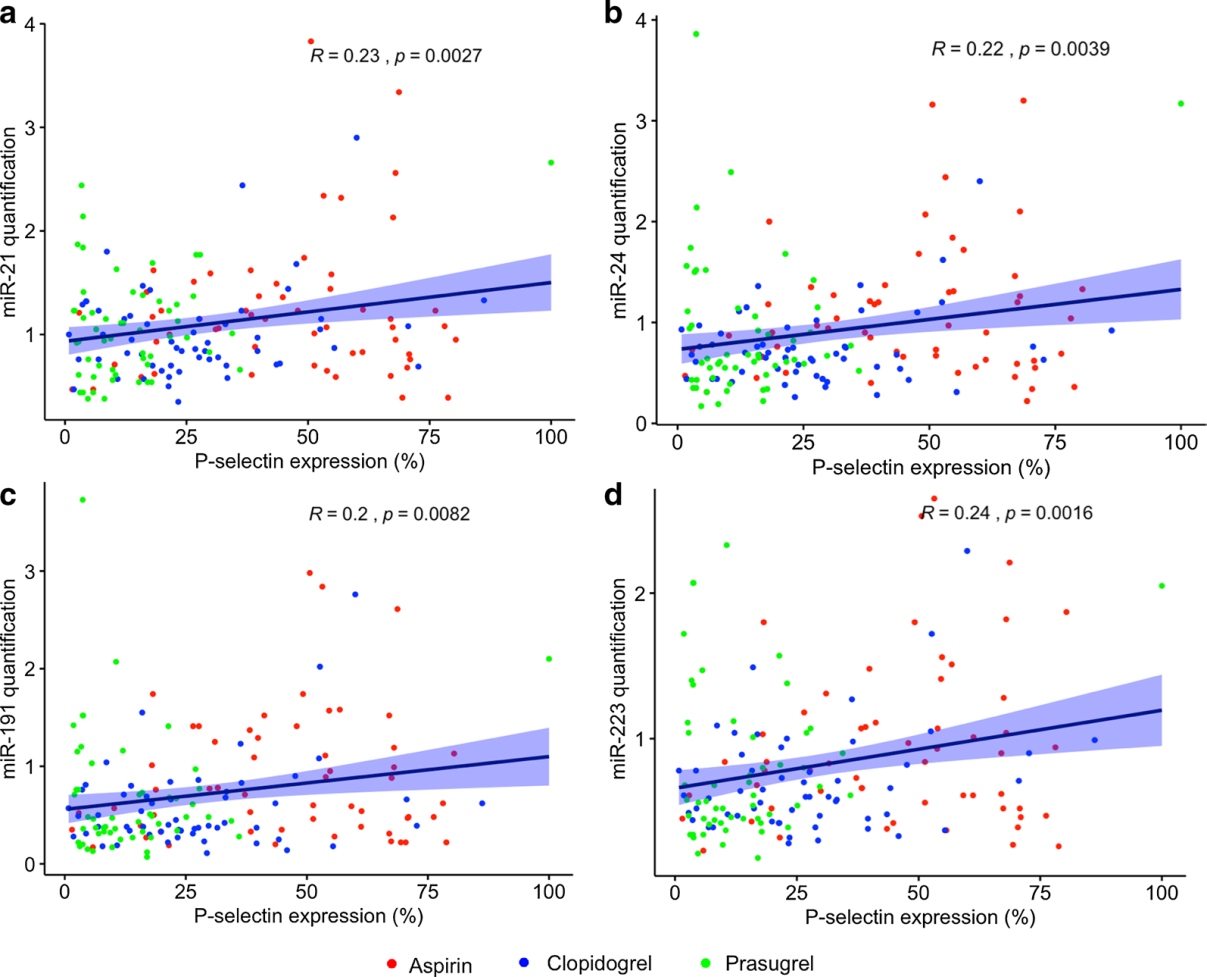
Correlation between platelet P-selectin expression, after stimulation with 30 μmol/L ADP, and relative quantity of **a** miR-21, **b** miR24, **c** miR-191 and **d** miR-223. Dark blue lines indicate those of best linear fit, light blue shading indicates 95% confidence interval. R and p values were produced by Pearson correlation analysis

Significant differences were seen between the treatments in circulating levels of miR-21, miR-24, miR-191, miR-197 and miR-223 (Table 4, Fig. 4). Post-hoc pairwise comparisons revealed significantly lower miRNA expression, when receiving prasugrel compared to aspirin, of miR-24 (p = 0.004), miR-191 (p = 0.019), miR-197 (p = 0.009) and miR-223 (p = 0.014), but not miR-21 (p = 0.10 (Table 5, Fig. 5). There were no significant differences in miRNA levels between aspirin and clopidogrel nor between clopidogrel and prasugrel.

**Table 4:** Levels of circulating miRNAs in patients with diabetes receiving aspirin, clopidogrel or prasugrel. Values shown are mean ± SD. P values were generated by one-way repeated measures ANOVA with Greenhouse–Geisser correction

miR	2^−(ΔΔCq)^ (mean ± SD)			p	Partial η^2^ (effect size)
	Aspirin	Clopidogrel	Prasugrel		
21	1.21 ± 0.67	1.04 ± 0.51	1.03 ± 0.47	0.028	0.065
24	1.05 ± 0.64	0.88 ± 0.70	0.75 ± 0.40	0.009	0.084
27b	1.09 ± 0.79	0.96 ± 1.22	0.75 ± 0.38	0.115	0.042
28	0.83 ± 0.54	0.65 ± 0.53	0.63 ± 0.60	0.083	0.046
93	1.06 ± 0.60	0.95 ± 0.74	1.01 ± 0.78	0.662	0.007
122	0.93 ± 0.82	0.98 ± 0.98	1.07 ± 1.00	0.278	0.023
126	0.93 ± 0.42	0.83 ± 0.47	0.80 ± 0.34	0.110	0.057
150	0.83 ± 0.34	0.81 ± 0.43	0.80 ± 0.36	0.777	0.004
191	0.87 ± 0.65	0.66 ± 0.62	0.59 ± 0.48	0.017	0.073
197	1.13 ± 0.68	0.97 ± 0.82	0.82 ± 0.45	0.019	0.072
223	0.93 ± 0.55	0.78 ± 0.50	0.70 ± 0.41	0.014	0.076
320	0.93 ± 0.46	0.82 ± 0.46	0.79 ± 0.40	0.087	0.045
451a	1.04 ± 0.65	0.95 ± 0.43	1.20 ± 0.99	0.165	0.034
486	0.96 ± 0.70	0.85 ± 0.45	1.11 ± 1.17	0.215	0.028

**Table 5:** Pairwise comparisons (with Bonferroni correction) for those miRNAs with significant differences between the three treatments on ANOVA

miR-	Aspirin vs. clopidogrel		Aspirin vs. prasugrel		Clopidogrel vs. prasugrel	
	Mean difference (95% CI)	p	Mean difference (95% CI)	p	Mean difference (95% CI)	p
21	0.17 (− 0.004 to 0.345)	0.058	0.177 (− 0.023 to 0.378)	0.100	0.007 (− 0.158 to 0.172)	> 0.99
24	0.165 (− 0.069 to 0.398)	0.262	0.295 (0.081 to 0.510)	0.004	0.131 (− 0.116 to 0.377)	0.586
191	0.208 (− 0.031 to 0.446)	0.108	0.272 (0.036 to 0.508)	0.019	0.064 (− 0.185 to 0.313)	> 0.99
197	0.153 (− 0.138 to 0.444)	0.596	0.311 (0.065 to 0.558)	0.009	0.158 (− 0.102 to 0.418)	0.416
223	0.146 (− 0.052 to 0.343)	0.220	0.226 (0.036 to 0.416)	0.014	0.08 (− 0.103 to 0.263)	0.852

*CI* confidence interval

### Correlation between miRNA detection and other markers

Platelet ADP-stimulated P-selectin expression correlated with circulating levels of miR-21 (R = 0.23, p = 0.003), miR-24 (R = 0.22, p = 0.004), miR-191 (R = 0.2, p = 0.008) and miR-223 (R = 0.25, p = 0.002), but not miR-197 (R = 0.12, p = 0.13) (Fig. 5, Additional file 1: Table S3). Conversely, there was a negative correlation between AA-induced platelet aggregation and levels of miR-24 (R = − 0.21, p = 0.004), miR-191 (R = − 0.20, p = 0.01), miR-197 (R = − 0.23, p = 0.002) and miR-223 (R = − 0.24, p = 0.002) but not miR-21 (R = -0.08, p = 0.30) (Additional file 1: Table S3). No significant correlations were observed between ADP- or collagen-induced platelet aggregation and circulating miRNA levels (Additional file 1: Table S3).

Of the fibrin clot parameters studied, there was a significant positive correlation between final clot turbidity and miR-21 (R = 0.22, p = 0.006, Additional file 1: Figure S3) but no other parameters, nor with other miRNAs (Additional file 1: Table 3). Subgroup analysis of levels miR-21, miR-24, miR-191, miR-197 and miR-223 by HRPR status revealed no significant differences between the groups (Additional file 1: Table S4).

### miR-126

Although levels of miR-126 have been linked to platelet and endothelial function in the general population [25], T2DM may be associated with lower detectable quantities [39]. P-selectin expression in response to ADP stimulation showed positive correlation with miR-126 (R = 0.27, p = 0.0004) (Additional file 1: Table S3, Additional file 1: Figure S4). On the other hand, we failed to show significant differences in quantification of miR-126 between the treatments and there was no evidence of a significant correlation between aggregation responses and miR-126 (Additional file 1: Table S2).

### Effect of presence or absence of cardiovascular disease

**Fig. 6:**
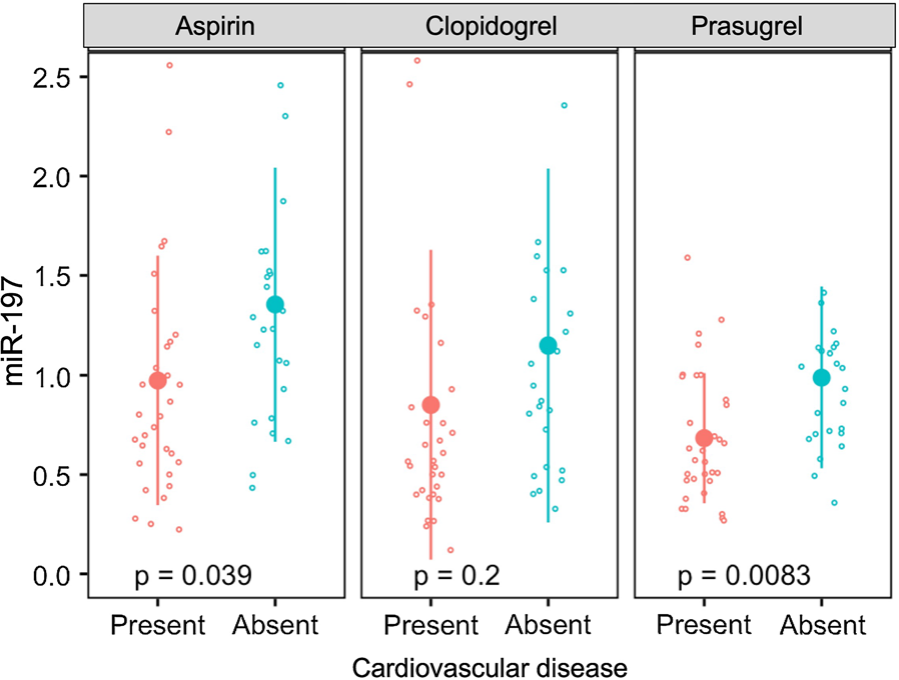
Relative quantification of circulating miR-197 in participants with and without a history of cardiovascular disease during the three treatment periods. Large dots and lines represent mean ± SD. p values were generated by t-tests

Subgroup analysis by presence (n = 32) or absence (n = 24) of a history of macrovascular atheromatous disease (history of coronary artery disease, cerebrovascular ischaemia or peripheral arterial disease) at enrolment revealed no significant differences between the subgroups in markers of platelet aggregation during each of the three treatment periods (Additional file 1: Table S5). Of the five miRNAs identified as having different expression profiles across the treatments, levels of miR-197 were significantly lower in those with cardiovascular disease compared to those without when receiving aspirin (0.97 ± 0.63 vs. 1.35 ± 0.69, p = 0.04) and prasugrel (0.68 ± 0.33 vs. 0.99 ± 0.46, p = 0.008), but not clopidogrel (0.85 ± 0.78 vs. 1.15 ± 0.89, p = 0.2) (Additional file 1: Table S5, Fig. 6). There were no significant differences between quantification of other miRNAs and cardiovascular disease state (Additional file 1: Table S5), including miR-126 (Additional file 1: Figure S6).

## Discussion

Patients with T2DM present particular challenges with regards to atherothrombotic protection. There is no current clear strategy of antiplatelet therapy for primary prevention in those with T2DM. For example, whilst some evidence suggests that aspirin therapy targeted by assessment of cardiovascular and bleeding risk may be of benefit [40], guidelines remain conflicted on the extent to which, if at all, aspirin therapy should be recommended in this situation [41, 42]. P2Y_12_ inhibitors are alternative antiplatelet agents to aspirin with the potential benefits of prolonged presence in the plasma that might overcome reduced aspirin effect due to high platelet turnover and avoiding gastric erosion [43]. Three orally-active drugs, clopidogrel, prasugrel and ticagrelor are commonly-available [6]. Monotherapy with clopidogrel offers only modest clinical benefits over aspirin in the setting of secondary prevention, but these may be amplified in those with T2DM [11]. Furthermore, the newer P2Y_12_ inhibitors ticagrelor and prasugrel are more potent and consistent in effect than clopidogrel [44]. Certainly, when given in combination with aspirin, ticagrelor and prasugrel offer net clinical benefit after ACS [6]. Additionally, there is evidence that potent P2Y_12_ inhibition may offer benefits in patients with T2DM and complications such as lower extremity arterial disease, in which ticagrelor improves microvascular flow, for example [45]. Although there has been concern over greater bleeding risk increasing the potency of P2Y_12_ inhibition, recent evidence suggests, for example, ticagrelor may have similar safety to clopidogrel in patients at high risk of bleeding, such as those who are elderly with ST-elevation myocardial infarction [46], and low-dose prasugrel appears to be of comparable safety to clopidogrel when used in triple therapy (aspirin, P2Y_12_ inhibitor and anticoagulant) for patients with atrial fibrillation undergoing percutaneous coronary intervention [47].

In this study, we have comprehensively characterised the effects of aspirin, clopidogrel and prasugrel when given as SAPT to patients with T2DM. Prasugrel provided the strongest effect on ADP-induced platelet aggregation, as did aspirin on AA-induced aggregation. Prasugrel also provided greater and more consistent inhibition of ADP-induced platelet aggregation when compared to clopidogrel, in agreement with previous studies on populations with and without diabetes receiving dual antiplatelet therapy [44, 48] and of studies of prasugrel vs. clopidogrel loading doses when given as SAPT [49]. In concert with this was the fact that there was a large reduction in the proportion of patients with HRPR when receiving clopidogrel vs. aspirin, and again when receiving prasugrel vs. either comparator. P-selectin expression after stimulation with ADP followed a similar pattern to the aggregation responses. Collagen-induced platelet aggregation, perhaps representing the best global assessment of effects on platelet macroaggregation, was more strongly inhibited by prasugrel than aspirin and clopidogrel, suggesting that prasugrel acts as the most potent antiplatelet drug of the three when used as monotherapy in patients with T2DM.

For the first time, we studied a panel of miRNAs in patients with diabetes receiving three different anti-platelet agents, showing that potent P2Y_12_ inhibition with prasugrel reduced detectable levels of miR-24, miR-191, miR-197 and miR-223 compared to aspirin. Although there were no significant differences between aspirin and clopidogrel nor clopidogrel and prasugrel, there did appear to be similar trends; thienopyridines reduced miRNA levels, an effect that was most pronounced with prasugrel. The known actions and associations of miRNAs are broad-ranging, but circulating levels of miR-21, miR-24, miR-197 and miR-223 are most strongly associated with platelets and platelet microparticles, along with miR-126 [26]. These, as well as miR-191, have been shown to be reduced by antiplatelet therapy in healthy volunteers, and some also in patients with cardiovascular disease [24, 25]. Our study, in individuals with T2DM, supports the association between plasma levels of these miRNAs and platelet function.

Whilst there were positive correlations between miRNA levels and ADP-induced platelet P-selectin expression, there was no evidence of a significant correlation with ADP or collagen-induced aggregation, and we observed negative correlations with AA-induced aggregation in some cases. P-selectin expression, which is known to be reduced by P2Y_12_ inhibitors but not aspirin [28], occurs when alpha granules fuse with the cell membrane upon platelet activation. These data suggest that plasma levels of miRNAs reflect primarily the tendency for platelet degranulation and indeed it has previously been shown that miRNAs may be involved in regulating degranulation [50]. This has not been reported before in individuals with diabetes receiving antiplatelet therapy and future research is required to understand the specific role of each of the miRNAs, which may help with risk stratification and/or uncover alternative therapeutic targets to control platelet activation in this population.

We saw lower plasma quantities of miR-197 in T2DM patients with known cardiovascular disease compared to those without, which is consistent with the previous finding in a general population cohort suggesting that lower miR-197 might be associated with increased risk of myocardial infarction [26, 51]. In contrast to previous findings in the general population, we did not see evidence of an association between elevated levels of miR-126 and presence of cardiovascular disease. Notably, however, studies have suggested that T2DM itself is associated with lower detectable quantities of miR-126 and therefore its prognostic significance as a vascular marker is potentially lost in the presence of diabetes and this may further explain the failure to demonstrate a treatment effect [39].

## Conclusion

In summary, our data suggest that prasugrel monotherapy is superior to either aspirin or clopidogrel in inhibiting platelet function in diabetes. From a translational perspective these findings could have the potential to be implemented in personalised treatment options for patients with T2DM and cardiovascular disease. Moreover, our miRNA results indicate that assessing response to antiplatelet therapy does not necessarily require fresh blood samples and tests can be conducted on miRNA measurements as biomarkers from stored acellular plasma samples. miRNA measurements may provide a platform to identify patients at greater risk of ischaemic heart disease relating to their platelet function. Based on these data and acknowledging that platelet activation is the central process in the development of atherothrombosis, a trial assessing the effects on clinical outcomes of prasugrel monotherapy may be warranted for the primary or secondary prevention of ischaemic heart disease in patients with T2DM. Also, further analysing the role of miRNA in predicting vascular outcome in individuals with diabetes may offer a tool to measure the clinical efficacy of antiplatelet agents.

## Supplementary information

**Additional file 1.** Additional figures and tables

## Data Availability

All data generated or analysed during this study are included in this published article and its Additional file 1.

## Abbreviations

AA: arachidonic acid
AU: absorbance units
ACS: acute coronary syndrome
CAPRIE: clopidogrel vs aspirin in patients at risk of ischaemic events
Cq: quantification cycle
FA: final aggregation
HRPR: high on-treatment residual platelet reactivity
MA: maximum aggregation
miRNA: micro ribonucleic acid
OD: once-daily
P2Y_12_: P2Y purinoceptor type 12 (platelet adenosine diphosphate receptor)
RT-qPCR: reverse transcription quantitative polymerase chain reaction
RNA: ribonucleic acid
SAPT: single antiplatelet therapy
T2DM: type 2 diabetes mellitus
