# Supplementary material for: Aspirin, clopidogrel and prasugrel monotherapy in patients with type 2 diabetes mellitus: a double-blind randomised controlled trial of the effects on thrombotic markers and microRNA levels
Source: Cardiovasc Diabetol. 2020 Jan 7;19:3. doi: 10.1186/s12933-019-0981-3 (PMC6945631; doi:10.1186/s12933-019-0981-3)
Supplement: Supplementary file 1 — Additional file 1. Additional figures and tables [file 12933_2019_981_MOESM1_ESM.docx]

**Additional material**

**Additional Table S1.** Platelet function at enrolment (receiving standard-of-care aspirin 75 mg OD) and after 14 days of study aspirin 75 mg OD, compared by paired t-tests. Data shown are mean ± SD.

|  | **Enrolment** | **End of study aspirin period** | **p** |
| --- | --- | --- | --- |
| Maximum aggregation responses (%) | | | |
| *Agonist* |  |  |  |
| AA 1mM | 5.1 ± 16.7 | 6.5 ± 18.8 | 0.63 |
| ADP 20 μmol/L | 75.7 ± 10.1 | 77.8 ± 8.4 | 0.17 |
| Collagen 2 μg/mL | 59.8 ± 21.0 | 62.3 ± 19.3 | 0.39 |
|  | | | |
| P selectin expression after ADP 30 μmol/L (%) | 45.1 ± 24.7 | 45.7 ± 21.6 | 0.85 |

**Additional Table S2.** Proportions of patients with high- (HRPR) and low-residual platelet reactivity (LRPR) compared between the three treatments. P values generated using chi-squared tests.

|  | Aspirin (n=56) | Clopidogrel (n=56) | Prasugrel (n=56) |
| --- | --- | --- | --- |
| HRPR | 56 (100%) | 30 (54%) | 3 (5%) |
| LRPR | 0 (0%) | 26 (46%) | 53 (95%) |

|  | Clopidogrel vs. Aspirin | Prasugrel vs. Aspirin | Prasugrel vs. Clopidogrel |
| --- | --- | --- | --- |
| Chi-squared | 33.9 | 100.6 | 31.3 |
| Relative risk of HRPR (95% CI) | 0.54 (0.41 – 0.66) | 0.05 (0.02 – 0.15) | 0.1 (0.03 – 0.28) |
| p value | <0.0001 | <0.0001 | <0.0001 |

**Additional Table S3.** Assessments of correlation between miRNA expression and variables relating to platelet function, coagulation and diabetes, generated using Pearson correlation coefficient tests. miRNA values were log-transformed prior to analysis. Significant correlations are highlighted in bold.

|  | Relative quantification | | | | | | | | | | | |
| --- | --- | --- | --- | --- | --- | --- | --- | --- | --- | --- | --- | --- |
| vs. | miR-21 | | miR-24 | | miR-126 | | miR-191 | | miR-197 | | miR-223 | |
|  | *R* | *P* | *R* | *p* | *R* | *p* | *R* | *p* | *R* | *p* | *R* | *p* |
| MA to ADP 20 μmol/L | 0.1 | 0.19 | 0.03 | 0.75 | 0.03 | 0.75 | 0.09 | 0.23 | 0.07 | 0.35 | 0.09 | 0.24 |
| MA to Collagen 2 μg/mL | -0.08 | 0.30 | -0.09 | 0.23 | -0.09 | 0.23 | -0.13 | 0.11 | -0.12 | 0.12 | -0.13 | 0.1 |
| MA to AA 1 mmol/L | -0.08 | 0.30 | -0.08 | 0.31 | -0.08 | 0.31 | **-0.20** | **0.010** | **-0.23** | **0.002** | **-0.16** | **0.04** |
| P selectin expression to ADP 30 μmol/L | **0.23** | **0.003** | **0.27** | **0.0004** | **0.27** | **0.0004** | **0.2** | **0.008** | 0.12 | 0.13 | **0.24** | **0.002** |
| Maximum absorbance | **0.22** | **0.006** | 0.061 | 0.44 | 0.061 | 0.44 | -0.016 | 0.84 | 0.088 | 0.27 | 0.013 | 0.87 |
| Lag time | -0.05 | 0.53 | 0.033 | 0.67 | 0.033 | 0.67 | -0.017 | 0.83 | 0.065 | 0.41 | -0.007 | 0.93 |
| Lysis time | 0.094 | 0.23 | 0.14 | 0.067 | 0.14 | 0.067 | -0.015 | 0.85 | 0.008 | 0.92 | 0.013 | 0.87 |

AA, arachidonic acid; MA, maximum aggregation

**Additional Table S4.** Relative quantification of miRNAs, maximum platelet aggregation responses and platelet P-selectin expression during periods of high- (HRPR) or low-residual platelet reactivity (LRPR). Data shown are mean ± SD. p values were generated with paired t-tests. AA, arachidonic acid.

|  | **HRPR (n=89)** | **LRPR (n=78)** | **p** |
| --- | --- | --- | --- |
| Relative quantification of miRNAs | | | |
| miR-21 | 1.16 ± 0.59 | 1.04 ± 0.50 | 0.16 |
| miR-24 | 0.97 ± 0.59 | 0.83 ± 0.62 | 0.14 |
| miR-191 | 0.80 ± 0.63 | 0.63 ± 0.55 | 0.065 |
| miR-197 | 1.02 ± 0.63 | 0.94 ± 0.72 | 0.43 |
| miR-223 | 0.87 ± 0.52 | 0.75 ± 0.46 | 0.1 |
|  | | | |
| Maximum aggregation responses (%) | | | |
| *Agonist* |  |  |  |
| ADP 20 μmol/L | 74.6 ± 9.7 | 35.8 ± 13.5 | <2.2 x10^-16^ |
| Collagen 2 μg/mL | 67.7 ± 20.4 | 61.7 ± 17.4 | 0.043 |
| AA 1 mmol/L | 29.8 ± 39.3 | 52.5 ± 32.7 | 7.3 x10^-5^ |
|  | | | |
| ADP-induced (30 μmol/L) P-selectin expression (%) | 40.9 ± 22.2 | 15.6 ± 14.2 | 1.86 x 10^-15^ |

**Additional Table S5.** Relative quantification of miRNAs, maximum platelet aggregation responses and platelet P-selectin expression in participants with and without a history of cardiovascular disease. Data shown are mean ± SD. p values were generated with paired t-tests. AA, arachidonic acid.

|  | **Drug** | | | | | | | | |
| --- | --- | --- | --- | --- | --- | --- | --- | --- | --- |
|  | **Aspirin** | | | **Clopidogrel** | | | **Prasugrel** | | |
|  | Cardiovascular disease | |  | Cardiovascular disease | |  | Cardiovascular disease | |  |
|  | Present (n=32) | Absent (n=24) | p | Present  (n=32) | Absent  (n=24) | p | Present  (n=32) | Absent  (n=24) | p |
| Relative quantification of miRNAs | | | | | | | | | |
| miR-21 | 1.29 ± 0.80 | 1.13 ± 0.43 | 0.34 | 0.97 ± 0.52 | 1.18 ± 0.51 | 0.14 | 0.92 ± 0.28 | 1.12 ± 0.59 | 0.14 |
| miR-24 | 1.00 ± 0.71 | 1.15 ± 0.54 | 0.36 | 0.88 ± 0.84 | 0.92 ± 0.52 | 0.81 | 0.73 ± 0.32 | 0.77 ± 0.43 | 0.76 |
| miR-191 | 0.78 ± 0.68 | 1.01 ± 0.62 | 0.19 | 0.68 ± 0.75 | 0.68 ± 0.43 | 0.99 | 0.58 ± 0.39 | 0.60 ± 0.55 | 0.89 |
| miR-197 | **0.97 ± 0.63** | **1.35 ± 0.69** | **0.04** | 0.85 ± 0.78 | 1.15 ± 0.89 | 0.2 | **0.68 ± 0.33** | **0.99 ± 0.46** | **0.008** |
| miR-223 | 0.86 ± 0.55 | 1.06 ± 0.57 | 0.19 | 0.75 ± 0.57 | 0.85 ± 0.47 | 0.51 | 0.67 ± 0.32 | 0.74 ± 0.43 | 0.53 |
|  | | | | | | | | | |
| Maximum aggregation responses (%) | | | | | | | | | |
| *Agonist* |  |  |  |  |  |  |  |  |  |
| ADP 20 μmol/L | 77.5 ± 8.4 | 78.1 ± 8.6 | 0.79 | 55.0 ± 17.5 | 60.4 ± 17.6 | 0.27 | 34.2 ± 15.1 | 33.8 ± 12.9 | 0.92 |
| Collagen 2 μg/mL | 61.6 ± 18.6 | 63.1 ± 20.4 | 0.77 | 71.6 ± 17.9 | 73.0 ± 18.6 | 0.77 | 59.9 ± 20.6 | 60.6 ± 15.9 | 0.89 |
| AA 1 mmol/L | 4.84 ± 14.7 | 8.67 ± 23.4 | 0.49 | 55.5 ± 37.9 | 71.5 ± 29.5 | 0.08 | 52.2 ± 32.5 | 53.1 ± 29.8 | 0.91 |
|  | | | | | | | | | |
| ADP-induced (30 μmol/L) P-selectin expression (%) | 47.6 ± 21.9 | 43.2 ± 21.6 | 0.46 | 27.3 ± 17.5 | 27.0 ± 21.0 | 0.95 | 14.0 ± 18.3 | 14.2 ± 9.1 | 0.97 |


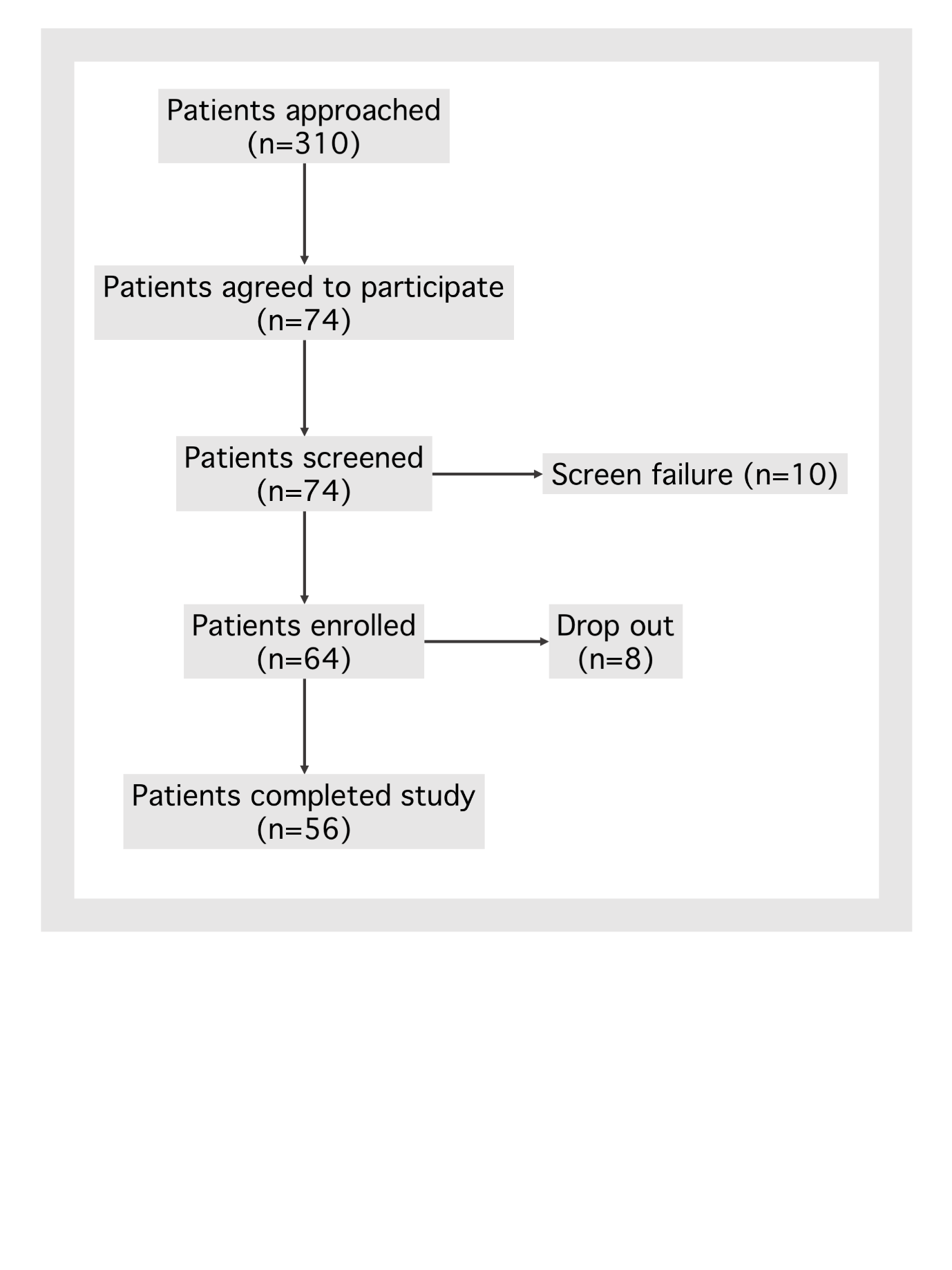


**Additional Figure S1.** Recruitment and follow up of study participants.


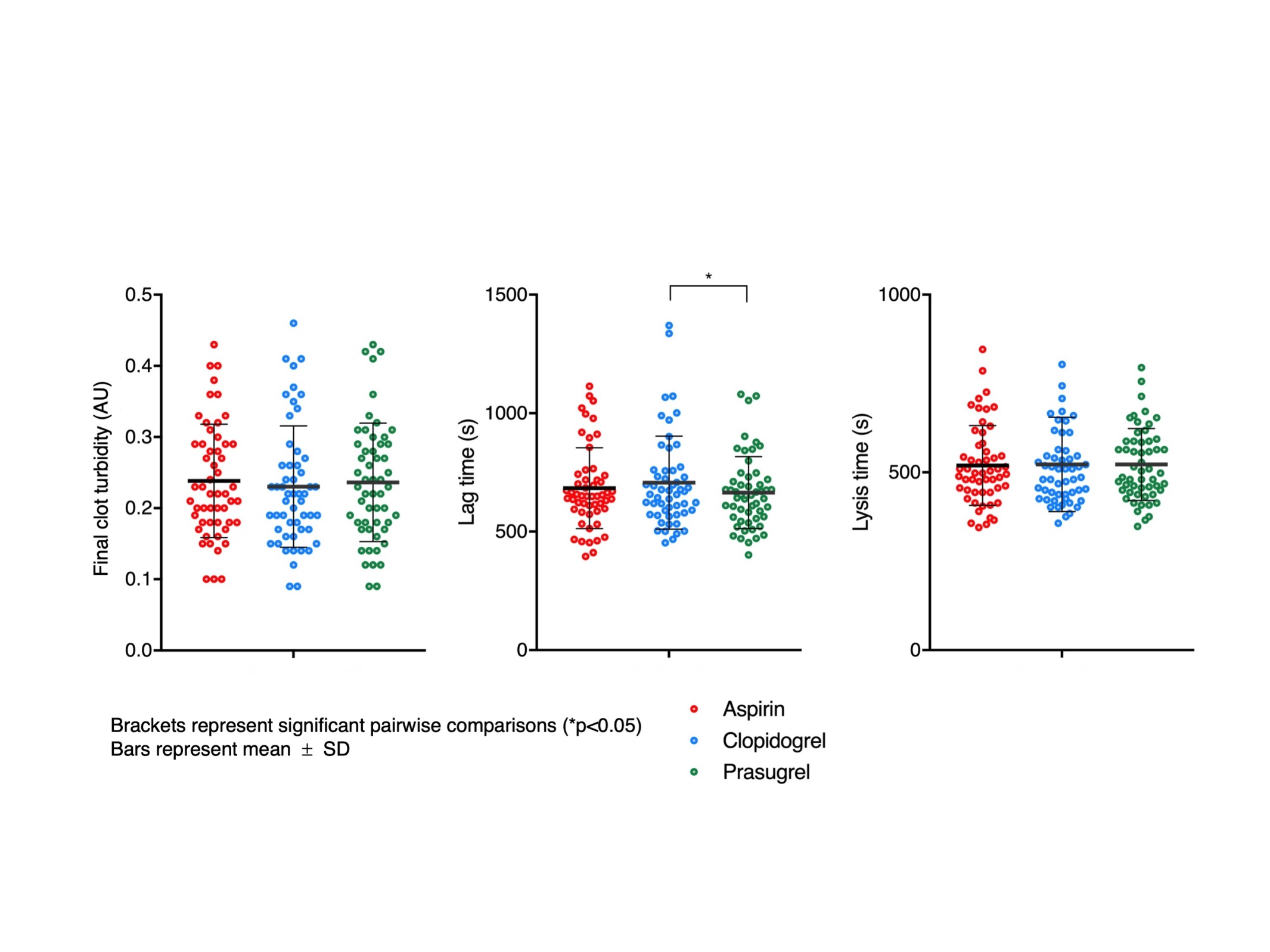


**Additional Figure S2.** Parameters of fibrin clot formation and lysis (A, final clot turbidity; B, lag time; C, lysis_2_ time; D, lysis_3_ time; E, lysis area under the curve) when receiving aspirin, clopidogrel or prasugrel. AU, absorbance units.

miR-21

Final clot turbidity (AU)

**Additional Figure S3.** Correlation of relative quantification of miR-21 with final clot turbidity. Dark blue line indicates that of best linear fit, light blue shading indicates 95% confidence interval. R and p values were produced by Pearson correlation analysis.

miR-126

**Additional Figure S 4.** Correlation of relative quantification of miR-126 with glycated haemoglobin A1c (HbA1c). Dark blue line indicates best linear fit, light blue shading indicates 95% confidence interval. R and p values were produced by Pearson correlation analysis.

**
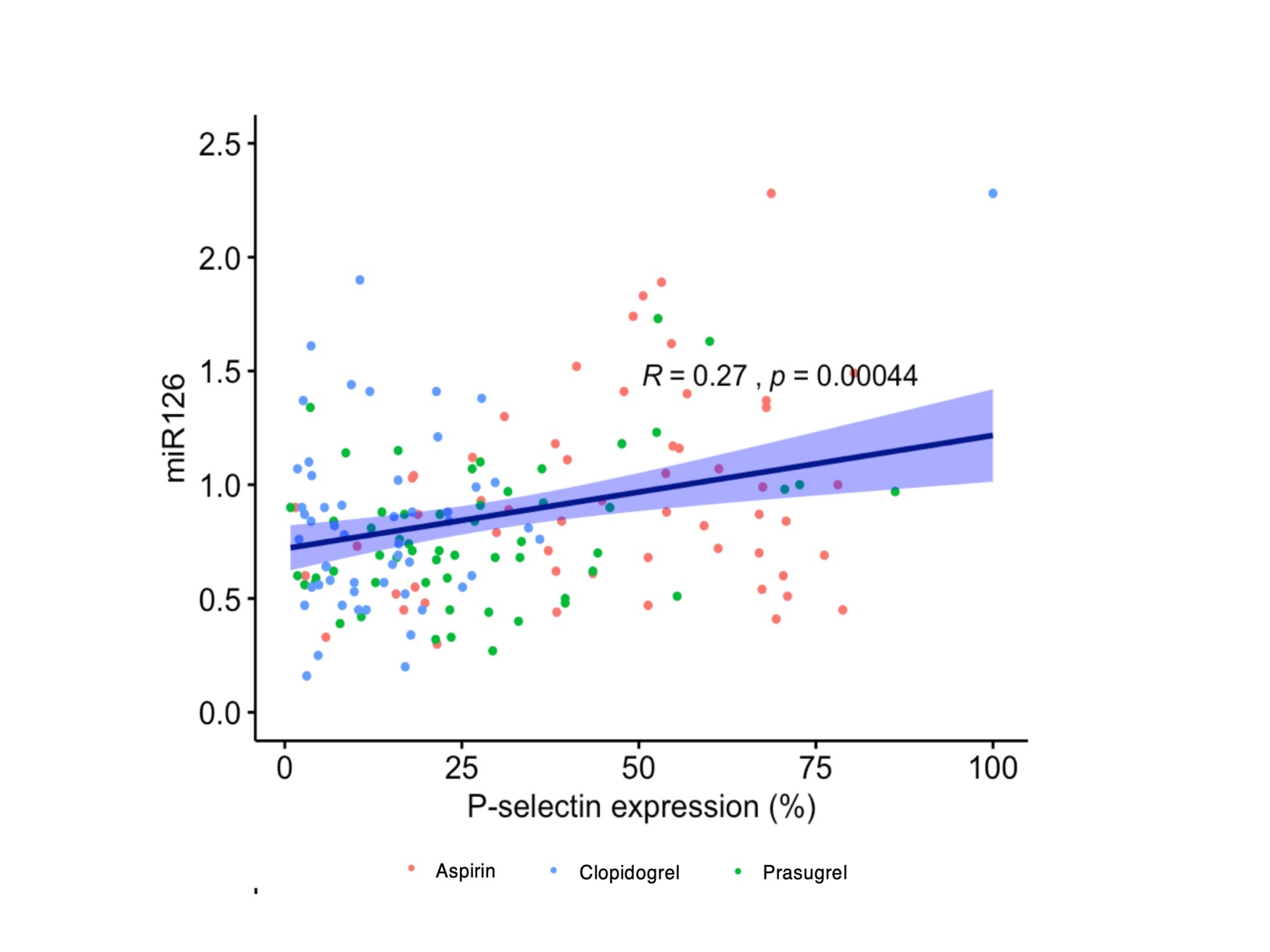
**

**Additional Figure S5.** Correlation of relative quantification of miR-126 with platelet P-selectin expression, after stimulation with 30 μmol/L ADP.


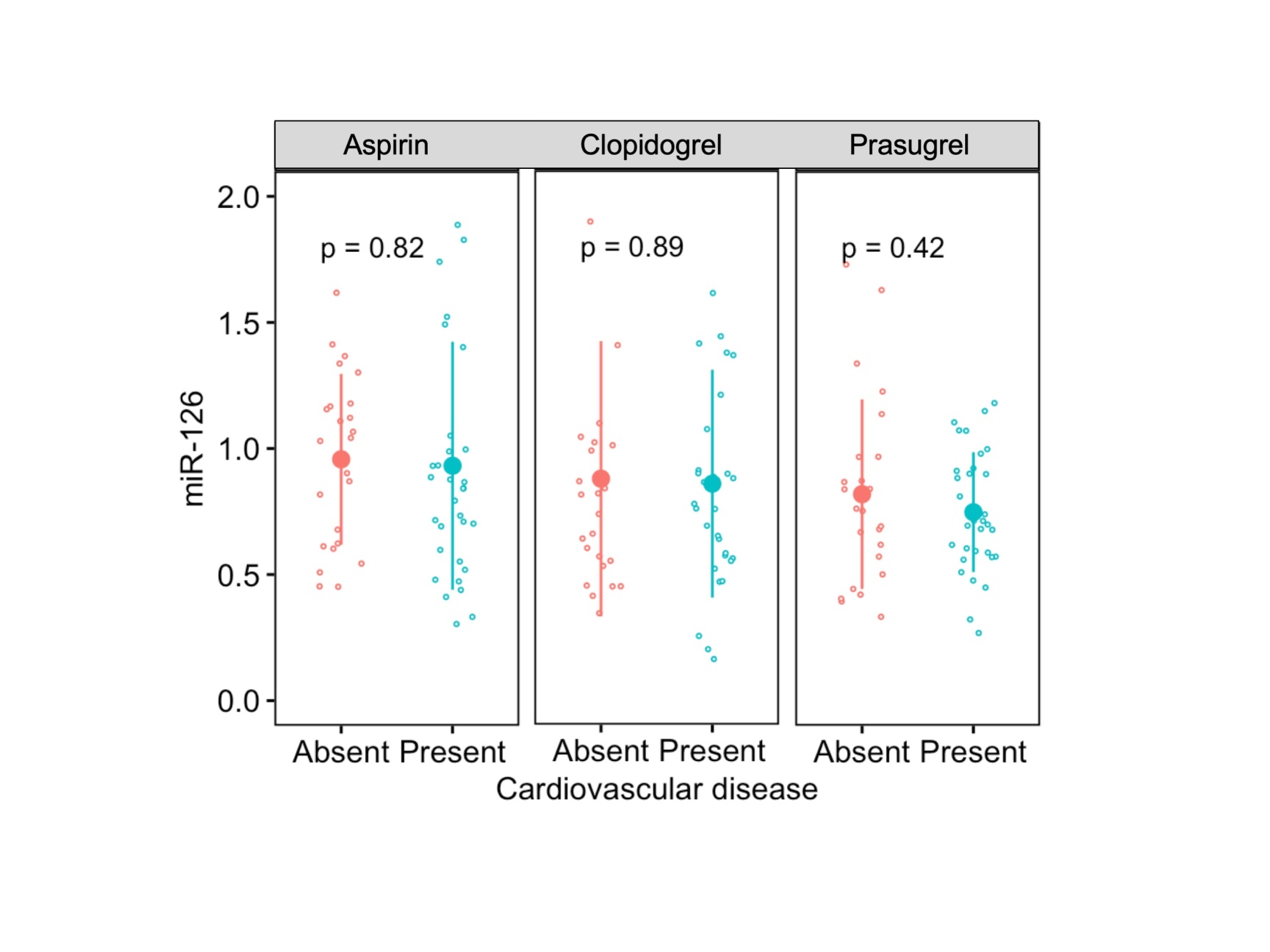


**Additional Figure S6.** Relative quantity of miR-126 in patients with diabetes mellitus when receiving aspirin, clopidogrel or prasugrel, stratified by presence or absence of known cardiovascular disease. P-values generated by t-tests.
